# How to Limit Interdialytic Weight Gain in Patients on Maintenance Hemodialysis: State of the Art and Perspectives

**DOI:** 10.3390/jcm14061846

**Published:** 2025-03-09

**Authors:** Maurizio Bossola, Ilaria Mariani, Camillo Tancredi Strizzi, Carlo Pasquale Piccinni, Enrico Di Stasio

**Affiliations:** 1 Nephrology, Dialysis and Transplantation Unit, Fondazione Policlinico Universitario A. Gemelli IRCCS, 00168 Rome, Italy; mauriziobossola@unicatt.it (M.B.); camillotancredi.strizzi01@icatt.it (C.T.S.); carlopasquale.piccinni01@icatt.it (C.P.P.); 2 Department of Translational Medicine and Surgery, Università Cattolica del Sacro Cuore, 00168 Rome, Italy; enricodistasio@unicatt.it; 3 Department of Basic Biotechnological Sciences, Intensive Care and Perioperative Clinics, Fondazione Policlinico Universitario A. Gemelli IRCCS, 00168 Rome, Italy

**Keywords:** hemodialysis, interdialytic weight gain, fluid management, thirst modulation, ultrafitration, digital health, precision medicine, patient adherence

## Abstract

**Background:** Interdialytic weight gain (IDWG), defined as the accumulation of salt and water intake between dialysis sessions, is a critical parameter of fluid management and a marker of adherence to dietary and fluid restrictions in hemodialysis patients. Excessive IDWG has been strongly associated with increased cardiovascular risk, including left ventricular hypertrophy, cardiac dysfunction, and cerebrovascular complications. Additionally, it necessitates more aggressive ultrafiltration, potentially compromising hemodynamic stability, impairing quality of life, and escalating healthcare costs. Despite international guidelines recommending an IDWG target of <4–4.5% of body weight, many patients struggle to achieve this due to barriers in adhering to dietary and fluid restrictions. This review explores the current state-of-the-art strategies to mitigate IDWG and evaluates emerging diagnostic and therapeutic perspectives to improve fluid management in dialysis patients. **Methods:** A literature search was conducted in PubMed/MEDLINE, Scopus, and Web of Science to identify studies on IDWG in hemodialysis. Keywords and MeSH terms were used to retrieve peer-reviewed articles, observational studies, RCTs, meta-analyses, and systematic reviews. Non-English articles, case reports, and conference abstracts were excluded. Study selection followed PRISMA guidelines, with independent screening of titles, abstracts, and full texts. Data extraction focused on IDWG definitions, risk factors, clinical outcomes, and management strategies. Due to study heterogeneity, a narrative synthesis was performed. Relevant data were synthesized thematically to evaluate both established strategies and emerging perspectives. **Results:** The current literature identifies three principal strategies for IDWG control: cognitive–behavioral interventions, dietary sodium restriction, and dialysis prescription adjustments. While educational programs and behavioral counseling improve adherence, their long-term effectiveness remains constrained by patient compliance and logistical challenges. Similarly, low-sodium diets, despite reducing thirst, face barriers to adherence and potential nutritional concerns. Adjustments in dialysate sodium concentration have yielded conflicting results, with concerns regarding hemodynamic instability and intradialytic hypotension. Given these limitations, alternative approaches are emerging. Thirst modulation strategies, including chewing gum to stimulate salivation and acupuncture for autonomic regulation, offer potential benefits in reducing excessive fluid intake. Additionally, technological innovations, such as mobile applications and telemonitoring, enhance self-management by providing real-time feedback on fluid intake. Biofeedback-driven dialysis systems enable dynamic ultrafiltration adjustments, improving fluid removal efficiency while minimizing hemodynamic instability. Artificial intelligence (AI) is advancing predictive analytics by integrating wearable bioimpedance sensors and dialysis data to anticipate fluid overload and refine individualized dialysis prescriptions, driving precision-based volume management. Finally, optimizing dialysis frequency and duration has shown promise in achieving better fluid balance and cardiovascular stability, suggesting that a personalized, multimodal approach is essential for effective IDWG management. **Conclusions:** Despite decades of research, IDWG remains a persistent challenge in hemodialysis, requiring a multifaceted, patient-centered approach. While traditional interventions provide partial solutions, integrating thirst modulation strategies, real-time monitoring, biofeedback dialysis adjustments, and AI-driven predictive tools represent the next frontier in fluid management. Future research should focus on long-term feasibility, patient adherence, and clinical efficacy, ensuring these innovations translate into tangible improvements in quality of life and cardiovascular health for dialysis patients.

## 1. Introduction

Interdialytic weight gain (IDWG), defined as the difference in weight between the end of one dialysis session and the beginning of the next, is widely recognized as a reliable indicator of fluid control in dialysis patients. It is, therefore, an indirect measure of patient adherence to prescribed fluid restrictions [1]. Numerous studies have demonstrated that excessive IDWG is associated with an increased risk of cardiovascular morbidity and mortality, including left ventricular hypertrophy and adverse cardiac and cerebrovascular events [2,3,4,5,6,7,8,9]. Furthermore, excessive IDWG often necessitates additional dialysis sessions, significantly reducing the quality of life and substantially increasing healthcare costs. The factors contributing to excessive IDWG primarily stem from barriers that hinder adherence to dietary and fluid restrictions. Although international guidelines recommend maintaining IDWG below 4–4.5% of body weight, various factors lead dialysis patients to exceed this target. The influence of the etiology of CKD, which may impact residual renal function and the fluid removal rate, should also be considered.

## 2. To Limit IDWG: State of the Art

**Figure 1:**
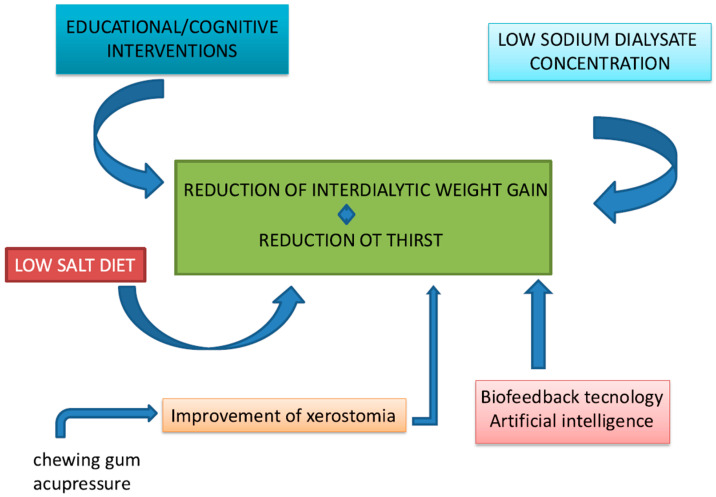
Strategies to reduce interdialytic weight gain and thirst in dialysis patients. A multifaceted approach—including educational interventions, dietary modifications, biofeedback technologies, and optimization of dialysate sodium concentration—contributes to improved fluid control. Addressing xerostomia through targeted measures, such as chewing gum and acupressure, further supports adherence to fluid restrictions.

Over time, several strategies have been proposed to enhance patient adherence and maintain IDWG within acceptable levels (Figure 1).

1. Educational/cognitive interventions, as well as counseling/behavioral and psychological/affective, have been employed to inform patients about their condition, encourage active participation in their care, and promote an appropriate lifestyle. These interventions aim to improve emotional and social aspects, fostering motivation. Studies have demonstrated that these approaches yield significant benefits in reducing IDWG in hemodialysis patients. The patient’s compliance is still the most important, as is the renal kidney function, on which the patient’s survival depends. However, these strategies also face challenges, including time constraints, high costs, and the need for patient compliance, as they require multiple training sessions, regular feedback, and homework. Even when good adherence is achieved, there remains a risk of relapsing [10,11,12,13]. Additionally, psychological interventions are limited by biases, such as the availability of qualified psychologists who may not be present in all dialysis centers. Moreover, the optimal duration of these interventions to achieve clinically meaningful effects remains unclear [12,13,14,15,16,17,18,19,20,21,22,23,24,25,26,27].
2. Another strategy to reduce IDWG involves lowering the sodium concentration in dialysis fluid. By reducing the sodium load in the dialysate, better sodium removal is achieved, resulting in lower overall sodium levels in circulation. This alleviates thirst, leading to reduced fluid intake between dialysis sessions. Moreover, sodium in the dialysis fluid is a source of sodium entry that can be adjusted independently of biosensors in monitors, mainly in HDF online, with a high infusion rate. Although the benefits of this approach have been well-documented in numerous studies, especially in Europe and the United States, it has also sparked considerable debate. Three recent systematic reviews reported conflicting results regarding the effects of dialysate sodium concentrations on IDWG [28,29]. Basile et al. found that most studies included in their analysis reported significantly higher IDWG in patients treated with high-sodium dialysate. However, their review of three interventional studies revealed no substantial differences in IDWG between treatment groups. Additionally, another study documented a reduction in total body weight in patients undergoing dialysis with both low (135 mmol/L) and high (140 mmol/L) sodium dialysate, although the difference between groups was not statistically significant [28]. In contrast, Dunlop et al. reported that low-sodium dialysate was associated with a significant reduction in IDWG compared to neutral or high sodium concentrations. However, the authors noted that the magnitude of this reduction was modest from a clinical perspective [29].

A critical concern regarding low-sodium dialysate involves the potential for adverse events, particularly intradialytic hypotension [28,30]. Nonetheless, a clinical review conducted by our group [31] found that most studies reported no significant differences in the frequency of hypotensive episodes between patients treated with low- or high-sodium dialysate [32,33,34,35,36,37,38,39]. The exception was a study by Marshall et al., which reported a higher incidence of hypotension in patients treated with low-sodium dialysate [30]. Several limitations were identified in the studies included in these reviews. First, the duration of studies varied considerably, ranging from one week to 12 months. Consequently, the medium- and long-term effects of reduced dialysate sodium concentrations remain largely unknown. Second, the definition of “low” sodium concentrations varied significantly across the studies reviewed, making it challenging to establish an “ideal” sodium concentration for clinical practice to effectively reduce IDWG. Third, the included studies exhibited high complexity and substantial heterogeneity.

3. An additional factor contributing to IDWG is excessive dietary salt intake, which increases thirst and subsequently leads to higher IDWG. According to international guidelines [40], the recommended daily sodium chloride intake for dialysis patients should not exceed 5 g (equivalent to 2.0 g [85 mmol] of sodium). For hypertensive dialysis patients, a stricter limitation of 2.5–3.8 g (1–1.5 g [43–65 mmol] of sodium) per day is advised. However, the average daily salt intake among dialysis patients remains significantly higher, ranging from 7.9 to 14.1 g/day [41,42,43,44,45,46,47,48]. Adherence to a low-sodium diet is often hindered by cultural, social, and economic factors. Moreover, the need to limit foods rich in phosphorus and potassium, which are often less palatable, may discourage adherence to a low-sodium diet [49]. Our systematic review and meta-analysis compared the effects of low-sodium diets versus normal or high-sodium diets, as well as the impact of nutritional counseling versus no counseling on IDWG in chronic hemodialysis patients [50]. The results revealed a significantly lower risk of experiencing IDWG > 2.5 kg among patients adhering to a low-sodium diet or receiving nutritional counseling compared to controls [51,52]. However, there are concerns about potential adverse effects associated with low-sodium diets, including pre-dialysis hyponatremia, malnutrition, and an increased risk of all-cause and cardiovascular mortality, that in the group of HD patients it is up to 33x the frequency [53,54,55,56]. Recent studies have also highlighted a correlation between low daily sodium intake and insufficient consumption of calories, proteins, minerals, trace elements, and vitamin B1 [53]. Hyponatremia can adversely affect the brain, heart, bones, musculoskeletal system, and immune system [57]. Overall, it seems that low-sodium diets or nutritional counseling for sodium reduction are effective strategies for achieving significant reductions in IDWG among chronic hemodialysis patients. Nevertheless, the limited availability of studies and the potential risks associated with these interventions underscore the need for further research. Prospective randomized trials with long-term follow-up are essential to clarify the effects of low-sodium diets, with particular attention to monitoring the development of hyponatremia, malnutrition, and other related complications.

In conclusion, the studies analyzed in our review clearly demonstrate that all these strategies—ranging from reducing sodium concentration in dialysate to implementing low-sodium diets and behavioral or psychological interventions—are effective solutions for reducing IDWG. However, their practical application and overall efficacy are limited by various challenges. Further research will be needed to provide greater clarity on the aspects already discussed, which remain the subject of ongoing investigation.

## 3. Thirst Is the Problem

Thirst is the physiological stimulus through which the body compensates for fluid loss and regulates the need for water. It is controlled by the central nervous system through neural and chemical signals [58]. The sensitivity to changes in water–electrolyte balance is heightened due to several factors, including the disruption of normal cellular function due to changes in the electrochemical gradient across the cell membrane and the reduction in blood pressure, which is necessary for the adequate transport of essential nutrients due to decreased blood volume. Consequently, an autonomic system (including changes in blood pressure, heart rate, diuresis, and natriuresis), a neuroendocrine system (release of oxytocin and ADH), and a behavioral system (enhancement of hunger and thirst) respond promptly to these changes. The kidneys play a central, albeit limited, role in this context due to their detoxifying activity and the presence of evaporative mechanisms that lead to continuous salt and water loss, even in the absence of excretion. Thus, fluid intake becomes an essential part of the response to osmolality changes [59].

Water–salt homeostasis relies on the balance between fluid and sodium intake and output, aiming to maintain circulatory integrity and normal osmolality of body fluids between 280 and 295 mOsm/kg [60]. The most important hormone in thirst regulation is vasopressin (ADH), a 9-amino acid peptide produced by neurons in the supraoptic and paraventricular nuclei of the hypothalamus and released from the posterior pituitary in response to increases in plasma osmolality, detected by osmoregulatory neurons [60].

Signals that induce the thirst mechanism include hypovolemia, hypotension, and angiotensin II, while those that inhibit it include hypervolemia, hypertension, and atrial natriuretic peptide (ANP) [58]. Angiotensin II is one of the primary responses to hypovolemia and hypotension, which occur in extracellular dehydration. It originates from the cleavage of hepatic angiotensinogen by renin and the subsequent cleavage of angiotensin I by the ACE enzyme. Among its actions are the promotion of water and salt consumption by acting on the cerebral subfornical organ (SFO), the promotion of peripheral vasoconstriction, and the stimulation of renal water reabsorption. Its effects have been mainly studied in rodents, but its role in humans appears less significant [59].

Baroreceptors, stretch-sensitive mechanoreceptors located in the heart and vessels, play a key role in monitoring blood pressure and, thus, in extracellular dehydration. Through their projections to the solitary tract nucleus via cranial nerves IX and X, they induce or inhibit thirst depending on the body’s needs. Additionally, they trigger the baroreflex to respond to acute blood pressure changes through the autonomic nervous system [59].

Through these regulatory mechanisms, two types of thirst can be distinguished: natremic-osmotic thirst and volumetric thirst. The former is induced by the activation of three regions: the SFO, the organum vasculosum of the lamina terminalis (OVLT), and the median preoptic nucleus (MnPO), which together form the lamina terminalis. Their activation leads to the activation of the paraventricular nucleus of the hypothalamus and, subsequently, the supraoptic nucleus, resulting in the production of ADH and the stimulation of thirst and water intake. Volumetric thirst, on the other hand, is triggered by the stimulation of volume receptors and baroreceptors located in the atria, pulmonary artery, vena cava, aortic arch, and carotid sinus due to a reduction in stretch forces in the vascular system caused by hypotension or hypovolemia [59,61,62].

Thirst sensation is also regulated by anticipatory signals to prevent potentially harmful imbalances due to excessive correction. There is a delay of about ten minutes between water intake and its complete absorption into the bloodstream. Consequently, the brain uses signals from the oropharynx to assess the intake and estimate how water will affect the water–electrolyte balance after absorption. One mechanism by which the oropharynx regulates water consumption seems to be based on the temperature of the liquid: cold liquids inhibit thirst neurons in the SFO more effectively than warm liquids. This may be an evolutionary trait linked to the fact that water intake tends to cool the oropharynx, allowing for an association between oral temperature and the post-ingestive effects of water consumption. Another anticipatory signal for thirst regulation occurs during eating: thirst is induced in advance to counterbalance the homeostatic burden imposed by ongoing food consumption. This phenomenon is so significant that if meal-related thirst is not followed by drinking, the phenomenon of “dehydration-induced anorexia” occurs. This involves a decrease in meal size but not meal frequency, indicating that it primarily affects meal termination [59].

Finally, an interesting concept is circadian thirst, in which, during sleep, sweating and urine production lead to a loss of fluids not associated with water intake, prompting the body to activate a compensatory mechanism by increasing fluid intake before sleep. From a physiological perspective, this behavior is linked to increased activity in the suprachiasmatic nucleus, which is connected to the OVLT [59,61,62,63].

### Focus on Dialysis Patients

Thirst and xerostomia are the main causes of poor adherence to fluid restriction in chronic hemodialysis patients. These patients are prescribed diets to reduce interdialytic weight gain (IDWG) and prevent fluid overload and hypertension, aiming for an IDWG value of less than 4.0–4.5% of dry body weight. Unfortunately, many patients have an IDWG greater than this value, with some experiencing IDWG as high as 10–20% [64].

Xerostomia refers to an uncomfortable dry mouth sensation that impairs the ability to taste food, swallow, and speak. It may be associated with halitosis and dental problems and predispose individuals to infections [65]. The literature data show that the percentage of HD patients suffering from xerostomia is high, ranging between 32.9% and 76.4% [66]. It has been demonstrated that hemodialysis patients exhibit reduced salivary function [67,68], and these alterations are often asymptomatic, possibly reflecting a more accelerated age-related decline in glandular function [69]. Factors contributing to the perception of dry mouth include anxiety, depression, and stress, along with the medications used to treat these conditions [70,71]. Other comorbidities, such as diabetes and heart failure, also contribute to the problem. In diabetes, hyperglycemia may lead to thirst and xerostomia [72], while in heart failure, thirst may be secondary to fluid restriction and sympathetic nervous system overactivation [73]. Recently, tangerine peel lemon glycerin extract oral spray has been used to mitigate micro-inflammation and malnutrition due to its antibacterial and anti-inflammatory properties, with improvement in the oral mucosal barrier and xerostomia [74].

Xerostomia is often accompanied by hyposalivation, defined as unstimulated salivary flow rates below 0.1 mL/min. However, this is not always the case. According to the literature, the association between xerostomia and normal salivation may be due to the coexistence of localized mucosal dehydration areas and areas with normal salivation. Studies aimed at understanding whether hyposalivation is linked to increased thirst sensation and IDWG in hemodialysis patients have shown weaker associations than expected. Furthermore, hyposalivation has been found to be associated with a high pre-dialytic sodium gradient, leading to cellular dehydration during dialysis sessions and reduced cellular capacity to produce fluids, including saliva [66].

Regarding the assessment of thirst in hemodialysis patients, two main tools are used: the Thirst Distress Scale (TDS) and the Dialysis Thirst Inventory (DTI). The former is a six-item instrument that measures thirst distress on a scale of 1–5, where 1 corresponds to “strongly disagree” and 5 to “strongly agree”. The items are as follows: (1) My thirst causes me discomfort; (2) My thirst bothers me a lot; (3) I am very uncomfortable when I am thirsty; (4) My mouth feels like cotton when I am thirsty; (5) My saliva is very thick when I am thirsty; (6) When I drink less, my thirst worsens [75]. The DTI is a seven-item questionnaire that quantifies perceived thirst. Each item uses a five-point Likert scale (never = 1, very often = 5). The scores are summed to provide a DTI score ranging from 7 (no thirst) to 35 (very thirsty). The items are as follows: (1) Thirst is a problem for me; (2) I am thirsty during the day; (3) My social life is influenced by my thirst; (4) I am thirsty before dialysis; (5) I am thirsty during dialysis; (6) I am thirsty after dialysis [76].

## 4. To Limit the IDWG: Perspectives

According to the available scientific literature, the appropriate application of educational/cognitive and counseling/behavioral strategies in planning intervention programs for hemodialysis patients (HDPs) could enhance patients’ abilities to manage their health conditions, yielding significant biological and psychological outcomes. A review by Zhianfar L et al. [77] examines how the psycho-educational approach employed in various studies aligns with the Therapeutic Patient Education (TPE) program recommended by the World Health Organization (WHO) [78]. This program is based on several key elements, including the following:

○ The presence of a psychologist in a multidisciplinary team comprising professionals such as nurses, dieticians, pharmacists, or physicians to develop patients’ skills and promote better adherence;
○ Integration of collective and individual practices, achieving more beneficial effects compared to a single approach;
○ Combination of biological/clinical outcomes (objective measures) and psychosocial criteria (subjective measures) to evaluate interventions targeting improvements in therapeutic adherence and lifestyle recommendations;
○ Continuous follow-up, accounting for short-, medium-, and long-term effects of interventions, as some results may decline over time [78].

However, it is evident that these principles are challenging to implement on a large scale, as demonstrated by the lack of psychologists in many dialysis centers and the difficulties posed by ensuring consistent follow-up, which requires the participation of patients who may not always be compliant. In this context, the psychological status of patients with chronic illnesses has been identified as a factor that can negatively influence adherence to therapeutic regimens and illness-related behaviors [79,80,81].

IT-based technologies have been recommended to support dietary self-monitoring in HDPs, thereby improving interdialytic weight gain (IDWG) and dietary biomarkers. Two systematic reviews [82,83] have suggested that technology can assist healthcare professionals in promoting better dietary adherence, for instance, by sending daily short text messages (SMS) to patients. Smartphone applications may further aid professionals in monitoring patients’ self-management behaviors and capabilities. The global expansion of digital technology underscores the need for further investment in planning IT-based interventions to enhance therapeutic adherence in patients with chronic diseases. An integrated patient-centered education and psychosocial support strategy has been proposed to comprehensively improve all dimensions of adherence to therapeutic and lifestyle recommendations, including prescribed medications, diet, dialysis, and fluid restrictions. These dimensions are often used as proxy measures for dialysis clinical outcomes and perceived quality of life (QOL) [84].

Several strategies for thirst reduction have been proposed: Besides dietary interventions to reduce salt intake (see paragraph 1), authors have used technical interventions targeting dialysis mechanisms, such as lowering the sodium concentration in the dialysate; pharmacological interventions (e.g., the use of ACE inhibitors, even if in patients without established end-stage kidney disease is almost irrelevant); salivary gland stimulation; consumption of cold water or ice chips; and reduction in intestinal sodium absorption through Tenapanor. To date, these interventions have been found to be either minimally effective or effective only in the short term [64].

Recently, other non-pharmacological interventions have been proposed, such as the use of chewing gum, frozen strawberries, acupressure, and a low-sodium diet continue to stand out but with limited benefits, which are only achieved through the combination of several non-pharmacological approaches [85].

1. Regarding *chewing gum* consumption, the potential mechanisms by which chewing gum may reduce thirst are
  - Salivation stimulation: Chewing stimulates saliva production, which may help alleviate the sensation of dry mouth and thirst. Indeed, an increase in saliva could reduce the perception of thirst, which is a common issue in hemodialysis patients due to fluid restrictions between dialysis sessions [86];
  - Psychological distraction: Chewing gum may serve as a psychological distraction, reducing awareness of thirst, which is often acute in dialysis patients due to the need to limit fluid intake between dialysis sessions [87];
    Unlike previously mentioned studies, Chen et al. found that the sensation of thirst was alleviated in the treated patient group, although no effect was observed on saliva production or IDWG in this population [85]. Similarly, Allida et al. demonstrated its short- and long-term effectiveness [88]. Dehghanmehr et al. found a significant difference between thirst and dry mouth and chewing sugar-free gum before and after the intervention [89]. Similarly, a significant reduction in thirst was observed by Bots et al. [90], Fan et al. [91], and Duruk and Eser [92]. Nonetheless, it is important to note that while chewing gum may provide temporary relief from thirst, it does not address the underlying issues related to fluid balance in dialysis patients.
2. *Acupressure***:** Keskin et al. reported increased salivation, a reduction in the severity of thirst on the visual analogue scale (VAS), and an enhanced quality of life for hemodialysis patients when applied at the CV-23 (Ren-23, Lianquan - "Corner Spring"), SJ-17 (SJ-17 (San Jiao-17, Yifeng - "Wind Screen"), and Kid-1 (Kidney-1, Yongquan - "Gushing Spring") points. In particular, they examined the effect of 15 min of acupressure three times a week for six weeks, considering an intervention group and a control group, finding significant differences in the mental component sub-dimension of the “Quality of Life” scale at both the first and sixth weeks [93]. These findings are supported by the study of Yang et al. [94], which demonstrated that acupressure improved the salivary flow rate and the mean thirst intensity, although no statistically significant difference was observed in pre- and post-program salivary flow rate.
  Acupuncture could improve thirst through several mechanisms:
  - Regulation of body fluids: Acupuncture stimulates specific points in the body that may influence fluid balance. It could help improve the equilibrium of body fluids, thereby reducing the sensation of thirst, which is often problematic for dialysis patients [95];
  - Effects on the nervous system: Acupuncture may have a positive impact on the autonomic nervous system, which is responsible for involuntary body functions such as thirst regulation. By stimulating certain points, acupuncture might reduce excessive thirst and improve the quality of life for dialysis patients [95];
  - Improvement of blood flow and fluid retention: Acupuncture could enhance blood circulation, reduce fluid retention, and improve kidney function. These effects might contribute to better fluid balance, thereby reducing the sensation of thirst [96].
  - Reduction in stress and pain: Acupuncture is also known for its effects in reducing stress and pain, and since dialysis patients may experience discomfort and frustration related to their condition, reducing stress could help minimize the need to drink excessively as an emotional response [97].

Since direct research on acupuncture for thirst in hemodialysis is limited, it may be useful to consider additional studies exploring the impact of acupuncture on fluid control and dialysis-associated symptoms, as well as its effects on the overall quality of life of patients.

3. *Frequency and duration of HD*: increasing the frequency of dialysis sessions and extending session duration are two promising strategies for optimizing fluid management and improving patient outcomes. Evidence highlights that more frequent dialysis regimens—such as daily or nocturnal sessions—consistently reduce interdialytic weight gain (IDWG) compared to the conventional thrice-weekly schedule [98]. Notably, findings from the Dialysis Outcomes and Practice Patterns Study (DOPPS) underscore the superior efficacy of frequent dialysis in controlling fluid overload, an improvement intricately tied to better cardiovascular health and reduced mortality [98]. The compelling results of the Frequent Hemodialysis Network (FHN) trial further emphasize the clinical advantages of increased frequency. Researchers reported that patients undergoing in-center hemodialysis six times per week experienced significant reductions in IDWG, improved blood pressure control, and better overall fluid balance compared to those receiving standard thrice-weekly treatments [99]. This is particularly critical given the robust association between elevated IDWG and adverse cardiovascular consequences, including left ventricular hypertrophy and heightened hospitalization rates [100,101]. Equally important is the role of session duration in fluid management. Prolonging dialysis sessions allows for a more gradual ultrafiltration process, effectively reducing the risk of intradialytic hypotension and minimizing complications associated with rapid fluid shifts [102,103]. Longer session lengths have been strongly associated with better blood pressure control, enhanced cardiovascular outcomes, and lower IDWG [98,104]. These outcomes highlight the potential of extended session duration to complement increased frequency in achieving optimal fluid management. Rather than viewing increased frequency or extended duration as isolated strategies, clinicians should consider an individualized approach that integrates both, tailored to the patient’s specific needs. For patients with high IDWG, frequent, shorter sessions might be more practical, whereas those prone to hemodynamic instability may benefit more from longer, less frequent sessions. By balancing these factors, it is possible to achieve a more patient-centered dialysis regimen that improves both immediate and long-term outcomes.
4. *Biofeedback technology*: the utilization of biofeedback systems in hemodialysis has emerged as a promising approach to assess and manage volume overload and interdialytic weight gain (IDWG) in patients undergoing this treatment. Biofeedback systems facilitate real-time monitoring of blood volume and other physiological parameters, allowing for tailored interventions that can mitigate these risks. One of the primary advantages of biofeedback systems is their ability to guide ultrafiltration (UF) rates based on continuous blood volume monitoring. This real-time feedback can help prevent excessive fluid removal, which is a common cause of intradialytic hypotension (IDH) [105,106]. Studies have shown that patients using biofeedback-guided UF experience fewer episodes of IDH compared to those receiving conventional hemodialysis [107,108]. A randomized controlled trial demonstrated that blood volume monitoring significantly reduced the frequency of symptomatic IDH, thereby improving overall patient stability during dialysis sessions [106]. Moreover, biofeedback systems can play a crucial role in managing IDWG, which is often a reflection of fluid retention between dialysis sessions. By providing real-time data on fluid status, biofeedback systems enable clinicians to make informed decisions regarding fluid removal and dietary recommendations, potentially reducing IDWG [109]. The study of Mohamed et al. found that patients who received biofeedback on their fluid status were better able to adhere to fluid restrictions, resulting in lower IDWG [110].

However, it is important to acknowledge the limitations and challenges associated with the implementation of biofeedback systems in clinical practice. While many studies demonstrate the efficacy of these systems, there are still gaps in the literature regarding their long-term impact on patient outcomes and the optimal integration into existing hemodialysis protocols [107,111]. Furthermore, the variability in individual patient responses to biofeedback interventions necessitates ongoing research to refine these technologies and establish standardized protocols for their use [107,111].

In conclusion, biofeedback systems represent a valuable advancement in the assessment and management of volume overload and interdialytic weight gain in hemodialysis patients. By enabling real-time monitoring and personalized treatment adjustments, these systems can improve patient outcomes, reduce the incidence of intradialytic hypotension, and enhance overall quality of life. As research continues to evolve in this area, the potential for biofeedback technology to transform hemodialysis practice remains significant.

5. *Wearable Devices and Artificial Intelligence Integration*: the impact of wearable devices on clinical outcomes in hemodialysis patients is a rapidly evolving area of research with significant implications for patient management and health outcomes. Wearable technologies have the potential to enhance patient engagement, facilitate self-management, and provide real-time data that can inform clinical decision-making. One of the primary benefits of wearable devices is their ability to monitor physiological parameters continuously, which is crucial for managing conditions such as fluid overload in hemodialysis patients. For instance, wearable technologies can track vital signs and fluid status, enabling patients to receive immediate feedback on their health status. This real-time monitoring can help patients adhere to fluid restrictions and dietary guidelines, which are essential for minimizing IDWG and preventing complications associated with fluid overload [112]. Studies have indicated that the use of wearable devices can lead to improved adherence to treatment protocols, resulting in better clinical outcomes, including reduced hospitalization rates and improved quality of life [113]. These devices utilize advanced sensors to assess various physiological parameters, including bioimpedance and thoracic fluid levels, which correlate strongly with fluid changes in the body [112,114]. For instance, a study demonstrated that a wearable bioimpedance device could effectively monitor fluid overload and provide feedback to patients, allowing for timely interventions [114]. This capability is particularly crucial as traditional methods often rely on periodic assessments in clinical settings, which may not capture fluctuations in fluid status between dialysis sessions [112]. Research has shown that wearable bioimpedance devices can effectively track changes in fluid volume, thereby facilitating better fluid management strategies [115,116]. Integration of mobile applications that track dietary intake and fluid consumption can further enhance patient engagement and adherence to prescribed regimens [115,117]. Bioimpedance measures the resistance of body tissues to electrical currents, providing insights into hydration levels and fluid overload [118]. Recent advancements in textile electrodes have improved the comfort and usability of these devices, enabling long-term monitoring without compromising patient mobility [118]. The ability to track fluid status continuously can help patients adhere to fluid restrictions and prevent complications associated with fluid overload, such as cardiovascular events. Moreover, the integration of artificial intelligence and digital health technologies into these wearable devices can enhance their functionality. AI algorithms can analyze data collected from patients to provide personalized recommendations for fluid intake and dietary adjustments, thereby promoting adherence to treatment protocols [112]. This is particularly relevant given that non-adherence to fluid restrictions is a common issue among hemodialysis patients, often exacerbated by factors such as thirst and psychological stress [49]. Wearable technologies can also incorporate features that support psychological well-being, such as reminders and motivational prompts, which can enhance adherence to fluid management strategies [117].

The materials used in these wearable devices are equally important. Advances in flexible and stretchable electronics enable the creation of lightweight, comfortable devices that can be worn for extended periods without discomfort [119]. The use of atomically thin materials in wearable sensors has shown promise for developing highly sensitive devices capable of detecting minute changes in physiological signals [120]. These materials can enhance the performance of wearable health monitoring systems, making them more effective in clinical settings. Moreover, the usability and user experience of wearable devices are critical for their acceptance and effectiveness. Researchers emphasize the importance of reliability and durability in wearable technology, as these factors significantly influence user satisfaction and adherence to treatment protocols [121]. Ensuring that patients receive adequate training and support when using these devices is essential to minimize technical issues and enhance the overall experience.

Wearable technologies can also contribute to better clinical decision-making. The data collected from these devices can be integrated into electronic health records, allowing healthcare providers to monitor patients’ conditions more effectively and make informed treatment decisions [112,122]. This integration can lead to more personalized treatment plans, as clinicians can tailor interventions based on real-time data regarding a patient’s fluid status and overall health. In conclusion, wearable devices hold considerable promise for improving clinical outcomes in hemodialysis patients. By enhancing patient engagement, facilitating self-management, and providing valuable data for clinical decision-making, these technologies can lead to better health outcomes and improved quality of life for individuals with chronic kidney disease. Ongoing research and development in this field are essential to fully realize the potential of wearable technologies in renal care.

Artificial intelligence plays a crucial role in enhancing the functionality of wearable devices. By employing machine learning algorithms, these devices can analyze vast amounts of data collected from patients, including physiological parameters and historical fluid intake patterns. This analysis enables the devices to provide real-time feedback and personalized recommendations for fluid management. For instance, AI algorithms can detect trends in fluid accumulation and alert patients when they exceed recommended fluid intake levels, thereby promoting adherence to treatment protocols. Additionally, AI can facilitate the automation of alarms and notifications, allowing for timely interventions when patients are at risk of fluid overload. Moreover, the incorporation of AI in wearable devices can enhance the overall user experience. By providing intuitive interfaces and personalized health education through mobile applications, these devices can empower patients to take an active role in managing their condition. Studies have shown that integrating health education with wearable technology can significantly improve disease literacy among dialysis patients, leading to better self-management and adherence to treatment [123]. The development of AI-powered wearable devices for fluid management in ESRD patients holds great promise for improving patient outcomes and enhancing the quality of care. By integrating advanced monitoring technologies, AI-driven analytics, and user-friendly interfaces, these devices can facilitate personalized treatment approaches that empower patients to manage their fluid status effectively.

In conclusion, limiting IDWG in patients on maintenance hemodialysis still remains a challenge for the physicians and the patients in the routine clinical practice. Much effort is needed to give patients an IDWG below 4–4.5% of body weight in terms of basic and clinical research in the future.
